# Hyponatremia in Cirrhosis and End-Stage Liver Disease: Treatment with the Vasopressin V_2_-Receptor Antagonist Tolvaptan

**DOI:** 10.1007/s10620-012-2276-3

**Published:** 2012-06-26

**Authors:** Paul Gaglio, Kwaku Marfo, Joseph Chiodo

**Affiliations:** 1 Division of Hepatology, Montefiore Medical Center and Albert Einstein College of Medicine, 111 East 210th Street, Rosenthal 2 Red Zone, Bronx, NY 10467 USA; 2 Medical Science Liaison, Otsuka America Pharmaceutical, Inc., Rockville, MD USA

**Keywords:** Arginine vasopressin, MELD score, Portal hypertension, Vasopressin receptor antagonists

## Abstract

Hyponatremia is common in patients with cirrhosis and portal hypertension, and is characterized by excessive renal retention of water relative to sodium due to reduced solute-free water clearance. The primary cause is increased release of arginine vasopressin. Hyponatremia is associated with increased mortality in cirrhotic patients, those with end-stage liver disease (ESLD) on transplant waiting lists, and, in some studies, posttransplantation patients. Clinical evidence suggests that adding serum sodium to model for ESLD (MELD) scoring identifies patients in greatest need of liver transplantation by improving waiting list mortality prediction. Hyponatremia is also associated with numerous complications in liver disease patients, including severe ascites, hepatic encephalopathy, infectious complications, renal impairment, increased severity of liver disease in cirrhosis, and increased hospital stay and neurologic/infectious complications posttransplant. Vasopressin receptor antagonists, which act to increase free water excretion (aquaresis) and thereby increase serum sodium concentration, have been evaluated in patients with hypervolemic hyponatremia (including cirrhosis and heart failure) and euvolemic hyponatremia (SIADH). Tolvaptan, a selective vasopressin V_2_-receptor antagonist, is the only oral agent in this class approved for raising sodium levels in hypervolemic and euvolemic hyponatremia. The SALT trials showed that tolvaptan treatment rapidly and effectively resolved hyponatremia in these settings, including cirrhosis, and it has been shown that this agent can be safely and effectively used in long-term treatment. Fluid restriction should be avoided during the first 24 h of treatment to prevent overly rapid correction of hyponatremia, and tolvaptan should not be used in patients who cannot sense/respond to thirst, anuric patients, hypovolemic patients, and/or those requiring urgent intervention to raise serum sodium acutely.

## Introduction

Hyponatremia is common in patients with cirrhosis and portal hypertension and is characterized by excessive renal retention of water relative to sodium as a result of reduced solute-free water clearance [1]. Hyponatremia may result from several factors related to cirrhosis and portal hypertension, the most prominent of which is increased release of arginine vasopressin (AVP; also known as antidiuretic hormone, or ADH). AVP release in portal hypertension is thought to occur via baroreceptor-mediated nonosmotic stimulation caused by a reduction in effective circulating volume, due in turn to arterial splanchnic vasodilation [1]. Other factors may include: (1) reduced production of solute-free water in association with reduced delivery of sodium to the distal tubule due to reduced glomerular filtration rate; and (2) increased sodium resorption in the proximal tubule.

Hyponatremia is associated with increased risk of mortality in patients with cirrhosis [2, 3], and in those on liver transplantation waiting lists [4]. A number of recent studies also have shown that hyponatremia is associated with greater severity of complications of cirrhosis, including difficult-to-control ascites [5, 6], and greater frequency of complications posttransplant, including neurologic disorders, renal failure, and infectious complications [7–9].

Conventional treatment of cirrhotic ascites includes sodium restriction, diuretic therapy, and large-volume paracentesis [10]. Fluid restriction in combination with orally administered aldosterone antagonists and loop diuretics is currently the primary approach to treating hypervolemic hyponatremia in cirrhosis. In some patients, these therapies may be ineffective by themselves or inappropriate. In recent years, a number of vasopressin receptor antagonists, which inhibit the effects of AVP and thereby increase free water excretion, have been evaluated in hyponatremic patients with cirrhosis or other causes of hypervolemic hyponatremia. The oral selective vasopressin V_2_-receptor antagonist tolvaptan is approved for treating hypervolemic and euvolemic hyponatremia, including that caused by cirrhosis [11, 12]. Experience with this agent is discussed in the following review.

## Consequences of Hyponatremia in Cirrhosis and End-Stage Liver Disease

### Pathogenesis

A disturbance in body water homeostasis is a common feature of advanced cirrhosis [1]. This disturbance, which is invariably associated with the presence of ascites, is characterized by an inability to adjust the amount of water excreted in the urine relative to the amount of water ingested. This leads to water retention and subsequent development of dilutional hyponatremia. Chronic elevation of AVP, which regulates both urinary concentration and excretion, can disrupt normal homeostatic correction of this hyponatremia. AVP interacts with three subtypes of G-coupled receptors: V_1A_, V_1B_, and V_2_ [10]. The latter receptors, which are found primarily in renal collecting duct cells, are coupled to adenylate cyclase, and their activation increases cyclic adenosine monophosphate (cAMP) levels. This, in turn, activates protein kinase A (PKA), which phosphorylates aquaporin-2 (AQP2) water channels localized in intracellular vesicles. Following phosphorylation, the vesicles move to the apical membrane, and the AQP2 channels are inserted into the membrane, promoting water reabsorption and, consequently, lowering plasma osmolality. Thus, V_2_ receptors represent an attractive therapeutic target in cirrhotic patients with hyponatremia.

### Prevalence

Recent data indicate that hyponatremia is present in as many as half of patients with cirrhosis and portal hypertension [5]. The Cirrhotic Ascites Patient Population Survey (CAPPS) collected data for 28 consecutive days on 997 patients with cirrhosis and ascites from 28 centers, including patients attending outpatient clinics as well as inpatients under the care of hepatologists [5]. Hyponatremia was found in 57 % of inpatients and 40 % of outpatients, including serum sodium levels ≤ 135 mEq/L in 49.4 %, ≤130 mEq/L in 21.6 %, ≤125 mEq/L in 5.7 %, and ≤120 mEq/L in 1.2 %. In a consecutive series of 126 cirrhotic patients admitted to the intensive care unit (ICU) of a tertiary care center, Jenq et al. [13] found serum sodium concentrations < 135 mEq/L in 53.2 % and ≤130 mEq/L in 28.6 %. Kim et al. found hyponatremia (≤135 mEq/L) in 20.8 % of 188 inpatients with cirrhosis [6]. Shaikh et al. [14] found serum sodium levels ≤ 135 mEq/L in 51.6 % of 217 cirrhotic patients at a single center.

The reported prevalence of hyponatremia in patients undergoing liver transplantation for end-stage liver disease (ESLD) varies. Among 213 consecutive patients with cirrhosis undergoing orthotopic liver transplantation at one center, Hackworth et al. [8] found that 42 % developed hyponatremia (serum sodium ≤ 130 mEq/L) at some point prior to transplantation, with 16 % remaining persistently hyponatremic at the time of transplantation. In evaluating data from the Organ Procurement and Transplantation Network for 2005 and 2006, Kim et al. [4] found that hyponatremia (<135 mEq/L) was present in 31 % of nearly 14,000 adults on the liver transplant waiting list. In an analysis of the NIDDK-LTD (National Institute of Diabetes and Digestive and Kidney Diseases Liver Transplantation Database) and MOLTO (Models for Optimal Liver Transplant Outcomes) databases, Yun et al. [15] found hyponatremia (serum sodium ≤ 134 mEq/L) in 31.3 % of 2,175 patients undergoing transplantation. In 241 consecutive patients undergoing transplantation at a single center, Londoño et al. [7] found hyponatremia (serum sodium < 130 mEq/L) at the time of transplantation in 8 % of patients.

### Outcomes in Cirrhotic Patients

The CAPPS survey provided the first large-scale assessment of the clinical correlates of hyponatremia in cirrhotic patients. In this population of 997 patients, hyponatremia (serum sodium < 135 mEq/L) was associated with severe ascites, large fluid accumulation rate, requirement for frequent large-volume paracentesis, and impaired renal function [5]. Although the frequency of complications was greatest in patients with serum sodium < 130 mEq, complications were still more common in those with levels of 130–135 mEq/L than in patients with normonatremia. Significantly greater risks for hepatic encephalopathy (odds ratio [OR] 3.40), hepatorenal syndrome (OR 3.45), and spontaneous bacterial peritonitis (OR 2.36) were observed in patients with serum sodium ≤ 130 mEq/L, and significantly greater risks of hepatic encephalopathy (OR 1.69) and hepatorenal syndrome (OR 1.75) were observed in those with serum sodium levels of 131–135 mEq/L compared with normonatremic patients.

**Fig. 1:**
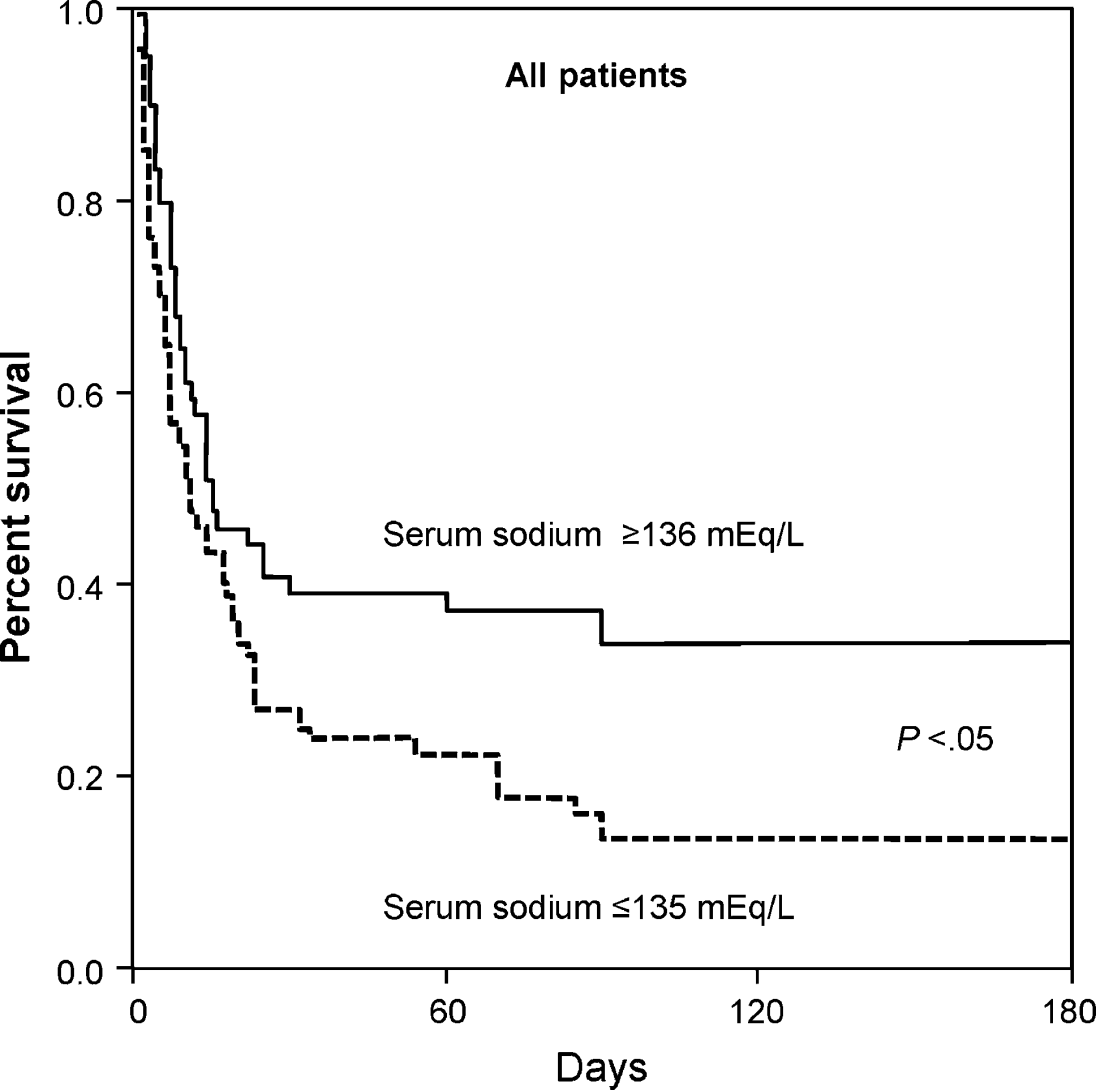
Six-month survival among 126 critically ill patients with cirrhosis. From Jenq et al. [13], reproduced with permission

In their study of 126 cirrhotic patients in an ICU, Jenq et al. found that hyponatremia was associated with increased frequency of ascites (OR 4.57, *P* < .001), hepatic encephalopathy (OR 2.38, *P* = .027), sepsis (OR 2.08, *P* = .048), and renal failure (OR 3.74, *P* = .001), as well as higher illness severity scores (based on MELD [model for end-stage liver disease], SOFA [Sequential Organ Failure Assessment], APACHE II [Acute Physiology and Chronic Health Evaluation II], and APACHE III rankings) [13]. It was also an independent predictor for both in- and out-of-hospital mortality, with in-hospital (73 vs. 56 %, *P* = .043) and 6-month mortality rates being significantly increased in hyponatremic versus normonatremic patients (Fig. 1). Most patients in this series had gastrointestinal bleeding, and hyponatremia was also significantly associated with mortality in this subgroup.

Other recent studies have shown that hyponatremia is an independent predictor of mortality in patients with ascites due to portal hypertension [16], and is significantly associated with increased severity of liver disease assessed by Child-Pugh and MELD scores [6], greater risk of spontaneous bacterial peritonitis and hepatic hydrothorax [6], and risk for hepatic encephalopathy [6, 17, 18].

With respect to hyponatremia and hepatic encephalopathy, there are no specific clinical features linking the two conditions; however, investigators have hypothesized that low-grade cerebral edema associated with hyponatremia may predispose cirrhotic patients to encephalopathy [19]. In this scenario, hyperammonemia in cirrhotic patients would lead to increased intracellular glutamine in astrocytes via the glutamine synthase pathway, raising intracellular osmolality and resulting in passage of fluid from the extracellular to the intracellular compartment. The resultant astrocyte swelling would then cause astrocyte dysfunction, primarily as a result of increased oxidative stress, thereby facilitating development of hepatic encephalopathy.

### Outcomes in Patients Awaiting Transplantation and Posttransplantation Patients

**Fig. 2:**
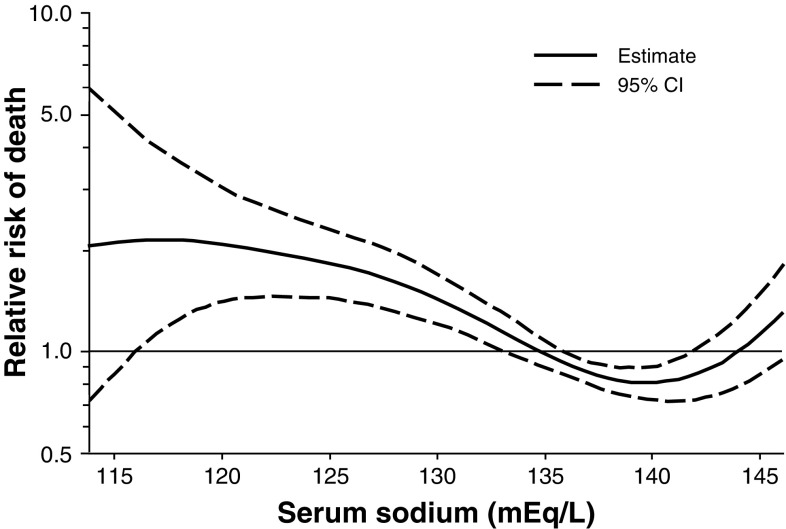
Relative risk of death (with 95 % confidence intervals [CI]) according to serum sodium concentration after adjustment for MELD score among 6,769 registrants in the Organ Procurement and Transplantation Network (2005 and 2006). From Kim et al. [4] reproduced with permission

Hyponatremia is associated with increased mortality in patients on the liver transplant waiting list. Data reported by Kim et al. [4] on 6,769 registrants in the Organ Procurement and Transplantation Network for 2005 and 2006 indicate that the serum sodium level and MELD score were significantly associated with mortality, with a hazard ratio of 1.21 per MELD point and 1.05 per 1-unit decrease in serum sodium level from 140 to 125 mEq/L (*P* < .001 for both). Decreases in serum sodium concentration were associated with an increased risk of death even after adjustment for MELD score (Fig. 2). The authors noted that a significant interaction between MELD score and serum sodium was observed, and that a revised MELD score utilizing a combination of the two factors in assigning transplantation priority might have resulted in avoidance of death in a sizable proportion of patients (see below for further discussion). In 296 patients referred for transplantation, Heuman et al. [20] found that MELD score, persistent ascites, and serum sodium level < 135 mEq/L were independent predictors of early mortality on multivariate analysis. MELD score was the sole independent predictor among patients with MELD scores > 21, whereas persistent ascites and low serum sodium were the only predictors among patients with MELD scores < 21; in the latter group of patients, serum sodium was an independent predictor whether analyzed as a continuous variable or as a categorical variable with a cutoff of 135 mEq/L.

In a recent study in the posttransplant setting, Yun et al. [15] found that serum sodium had no impact on 90-day survival among 2,175 primary orthotopic liver transplant recipients. Patients with severe hyponatremia (serum sodium < 125 mEq/L) had longer ICU and hospital stays, and the incidence of central pontine myelinolysis, although low, was correlated with serum sodium levels. Hackworth et al. [8] also found no difference in 180-day mortality rate among patients with hyponatremia (≤130 mEq/L) at transplant, those with hyponatremia that resolved before transplant, and normonatremic patients in a retrospective analysis of 213 transplant recipients. However, patients with resolved or current hyponatremia had increased time until discharge (*P* < 0.01), longer ICU stay (*P* = 0.03), and higher rates of delirium (*P* < 0.01), acute renal failure (*P* < 0.01), acute cellular rejection (*P* = 0.03), and infection excluding sepsis and pneumonia (*P* = 0.02) than never-hyponatremic patients, and patients with resolved hyponatremia were more likely to be discharged at 3 weeks than those with persistent hyponatremia (*P* = 0.04). Other investigators have found an association of hyponatremia with mortality in the posttransplant setting. Weissmüller et al. [9] found that a MELD score > 30 (OR 4.17), serum sodium < 130 mEq/L (OR 2.07), and pretransplant hemodialysis (OR 2.35) were the main predictors of death among 462 patients undergoing liver transplantation. Similarly, in 241 consecutive transplant patients, Londoño et al. [7] found a significantly lower 3-month survival rate (84 vs. 95 %, *P* < 0.05) in hyponatremic patients (serum sodium < 130 mEq/L), as well as increased risk for neurologic disorders (OR 4.6), renal failure (OR 3.4), and infectious complications (OR 2.7). Survival was similar in hyponatremic and normonatremic patients after 3 months. In addition, a retrospective study by Boin et al. [21] in 318 consecutive patients undergoing liver transplantation showed that hyponatremic patients (serum sodium ≤ 130 mEq/L) had significantly reduced survival (*P* = 0.04). Fukuhara et al. [22] found that MELD score (*P* = 0.03) and hyponatremia (serum sodium ≤ 130 mEq/L; *P* = 0.005) were predictive of short-term graft survival, and hyponatremia was a significant predictor of sepsis (*P* < 0.001), renal dysfunction (*P* < 0.001), and encephalopathy (*P* = 0.026) in 134 consecutive patients undergoing living donor liver transplantation.

## Addition of Serum Sodium Level to MELD Scoring

**Fig. 3:**
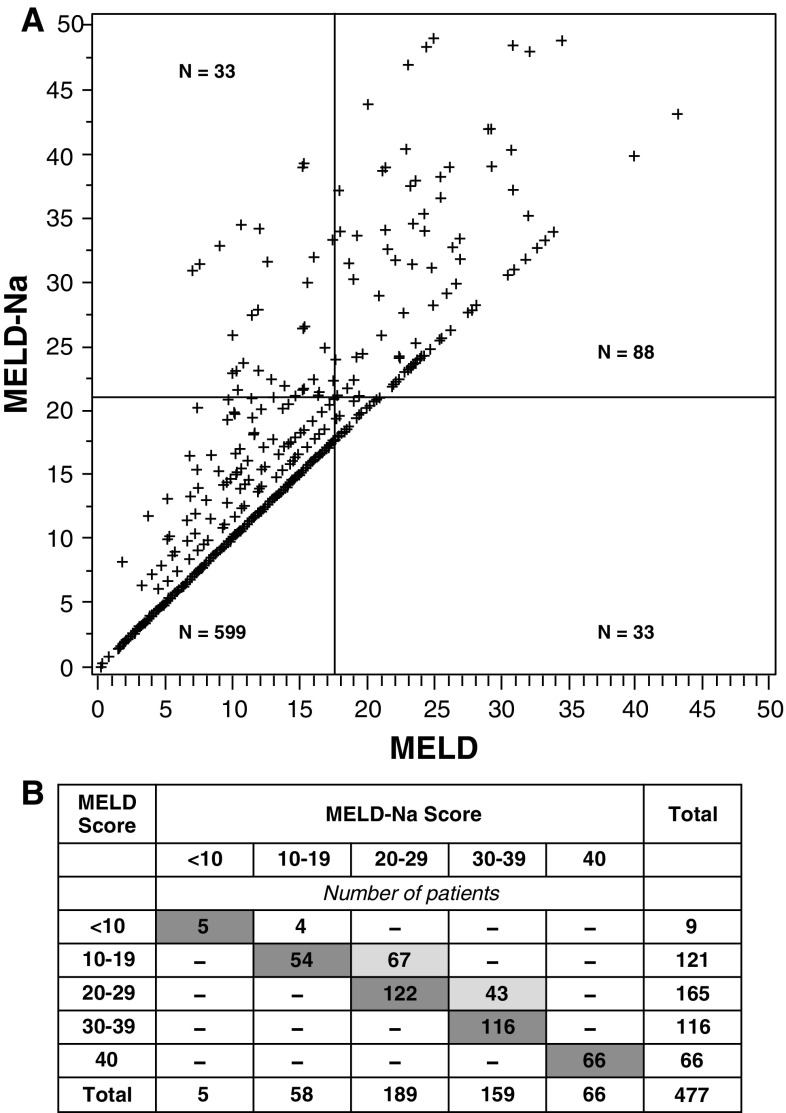
**a** Correlation of MELD score and MELD-Na score calculated by Biggins et al. showing change in transplant allocation priority. The *bisecting lines* represent 121 transplantations performed within 6 months of listing. The 33 patients in the *upper left* quadrant would have been favored by MELD-Na scoring over those in the *lower right* quadrant, who are favored by use of MELD alone. From Biggins et al. [23] reproduced with permission. **b** Distribution of MELD scores and Meld-Na scores calculated by Kim et al. for 477 patients who died on the transplant waiting list. *Dark shaded* cells indicate patients with similar MELD and MELD-Na scores. *Light shaded* cells represent patients with MELD-Na scores that were higher than their MELD scores and in a range that may have resulted in their selection for transplantation. The probabilities of receiving a transplant were 18.5 % for MELD scores of 10–19, 58.4 % for scores of 20–29, and 70.4 % for scores of 30–39. According to these percentages, 32 more patients may have received transplants if MELD-Na scoring had been used, potentially preventing death in 7 % of those who died on the waiting list. From Kim et al. [4] reproduced with permission

Considerable evidence suggests that addition of serum sodium level to MELD scoring better identifies patients in greatest need of liver transplantation by improving the prediction of waiting list mortality. Biggins et al. [23] developed a new scoring system to adjust MELD based on serum sodium (MELD-Na score: MELD + 1.59 (135 − serum sodium) for maximum and minimum sodium concentrations of 135 and 120 mEq/L, respectively). MELD-Na scores of 20, 30, and 40 were associated with a 6-month risk of death of 6, 16, and 37 %, respectively. It was estimated this scoring system would alter allocation of 27 % of grafts to patients who would have otherwise continued to wait to be transplanted (Fig. 3a). Similarly, in a study of 266 cirrhotic patients on the liver transplantation waiting list, MELD plus hyponatremia (≤130 mEq/L) predicted mortality significantly better than MELD alone (*P* = 0.006) [24]. Risk of death across all MELD scores was greater for patients with, versus without hyponatremia. In their study of patients awaiting transplantation, Kim et al. [4] found a significant difference in the c-statistic for ranking patients according to risk for death for a MELD-Na index (MELD − serum sodium − [0.025 × MELD × (140-sodium)] + 140, for sodium level between 125 and 140 mEq/L) versus the standard MELD index (0.883 vs. 0.868, *P* < 0.001). Comparison of use of the MELD-Na index and the MELD index among 477 patients who died within 90 days of transplant registration in 2006 showed that scores were similar using the two indices for 363 patients, and that differences between scores were sufficiently large for 110 (23 %) such that use of the MELD-Na score might have altered prioritization for graft allocation. With use of the MELD-Na score, it was estimated that 7 % of the deaths during this period might have been prevented (Fig. 3b).

## Clinical Presentation of Hyponatremia

The signs and symptoms of hyponatremia are primarily related to central nervous system dysfunction due to movement of water from intravascular spaces into brain cells, with resultant cerebral edema [25]. Initially, adaptive mechanisms promote the efflux of electrolytes (sodium, potassium, chloride) from brain cells, allowing for rapid adaptation. Subsequently, the extrusion of organic osmolytes (primarily glutamate) promotes adaptation at a slower pace. Both mechanisms serve to return water to intravascular compartments and limit the swelling of brain tissue [10]. The rapid adaptations are typically completed in approximately 48 h. Hyponatremia is classified as acute if it develops within 48 h or as chronic if it develops over a period exceeding 48 h [26, 27].

A number of factors determine the clinical presentation of hyponatremia, including the rate, extent, and overall timing of serum sodium decreases. The majority of patients with serum sodium levels greater than 125 mEq/L are asymptomatic as a result of the brain’s adaptive mechanisms; neurologic symptoms are more common when serum sodium falls below this value [25]. Rapid serum sodium decreases to 125–130 mEq/L in patients who had previously been asymptomatic may also produce symptoms. Symptoms commonly associated with hyponatremia include headache, nausea, vomiting, anorexia, muscle cramps, lethargy, restlessness, irritability, and disorientation, and are typically nonspecific. Rapid declines in serum sodium, which can overwhelm the brain’s adaptive mechanisms, or decreases to ≤120 mEq/L may produce more serious complications, including seizures, permanent brain damage, coma, brain stem herniation, respiratory arrest, and death [10, 25]. Low serum sodium levels, even in the absence of accompanying symptoms, raise the risk for development of more severe, symptomatic hyponatremia [28].

## Management of Hypervolemic Hyponatremia in Cirrhosis/ESLD

### Symptomatic Hyponatremia

Patients with acute hyponatremia (hyponatremia that develops in ≤48 h) are at risk for developing neurologic impairment and, consequently, require prompt correction of serum sodium levels; this is typically accomplished via administration of a hypertonic (3 %) saline infusion. It should be noted that there have been no dedicated studies to assess the effectiveness of this strategy, which potentially raises the risk of ascites and edema. In patients with chronic hyponatremia (hyponatremia that develops over a period > 48 h), the benefits of correction should be weighed against the risk of developing osmotic demyelination, a process associated with too-rapid normalization of serum sodium. Thus, serum sodium correction should be performed in a controlled manner.

The adaptive mechanisms that control brain swelling during development of chronic hyponatremia (onset in >48 h) can also make the brain susceptible to injury in response to overly rapid increases in serum sodium [10]. As serum sodium increases, the brain recaptures lost osmolytes, but at a slower rate than they were lost during the development of hyponatremia. When serum sodium is raised too rapidly, water may move from brain cells into the intravascular spaces, leading to cell shrinkage, and, ultimately, to osmotic demyelination. Although the specific mechanism has not yet been elucidated, investigators have suggested that myelin-producing oligodendrocytes may be more vulnerable to shrinkage than other types of brain cells, or that they may be preferentially damaged by inflammatory cells and mediators entering the brain after the blood–brain barrier is disrupted by shrinkage of vascular endothelial cells [29]. Thus, symptomatic patients may improve at first with rapid correction of serum sodium [30]; over the subsequent days, however, progressive neurologic deficits may emerge, and some of these may be permanent.

To avoid osmotic demyelination, serum sodium should be corrected in a controlled fashion: <10–12 mEq/L in 24 h and <18 mEq/L in 48 h [10, 30, 31]. For patients particularly susceptible to osmotic demyelination (e.g., those with severe malnutrition, alcoholism, or advanced liver disease), a slower rate of correction should be used. Because the brain’s adaptive mechanisms typically take 48 h to manifest, they are often incomplete in patients with acute (<48 h) onset of hyponatremia [32]. As a result, rapid correction of serum sodium is less likely to cause osmotic demyelination in these patients. When the timing of hyponatremia onset is unclear, it is prudent to assume chronic onset and avoid overly rapid elevations in serum sodium.

### Asymptomatic Hyponatremia

**Fig. 4:**
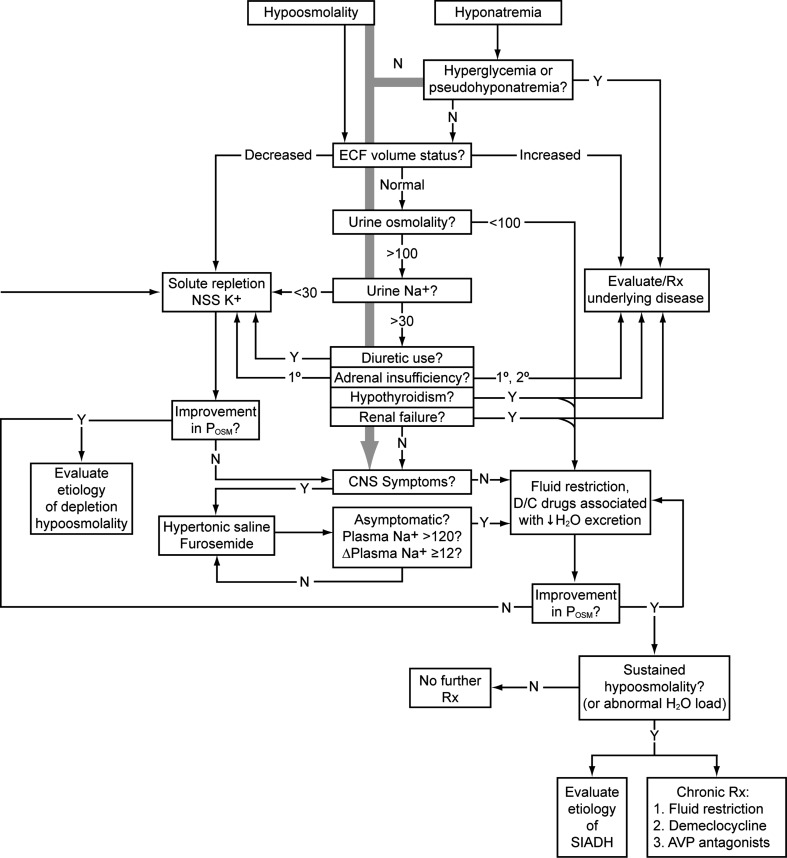
Algorithm for evaluation and treatment of hypo-osmolar patients. The *grey arrow* running down the center emphasizes that the presence of central nervous system dysfunction due to hyponatremia should always be assessed immediately, so that appropriate therapy can be started as soon as possible in symptomatic patients, even while the outlined diagnostic evaluation is proceeding. Values for osmolality are in mOsm/kg H_2_O, and those referring to serum sodium concentration are in mEq/L.* Δ *change (in concentration),* 1°* primary,* 2°* secondary,* AVP* arginine vasopressin,* CNS* central nervous system,* D/C* discontinue,* ECF* extracellular fluid,* N* no,* NNS* normal (isotonic) saline solution,* P*_osm_ plasma osmolality,* Rx* treat/treatment,* SIADH* syndrome of inappropriate antidiuretic hormone secretion,* Y* yes; (modified from Verbalis (2009) Hyponatremia and hypo-osmolar disorders. This chapter was published in: Greenberg A, Cheung AK, Coffman T, et al., eds. Primer on Kidney Diseases, 5th ed. Philadelphia: Saunders; 52–59)

Currently, there are no specific guidelines for the treatment of asymptomatic hyponatremia in cirrhosis. A general algorithm for the assessment and treatment of hyponatremia is presented in Fig. 4. As noted previously, fluid restriction in combination with orally administered aldosterone antagonists and loop diuretics is currently the primary approach to treating hypervolemic hyponatremia in cirrhosis. Fluid restriction typically produces only transient benefits, however, as restriction to 500 mL is generally required for benefits to be maintained [33]. In patients resistant to fluid restriction/diuretic therapy, pharmacologic agents such as demeclocycline—which is relatively contraindicated due to a high incidence of nephrotoxicity—and the vasopressin receptor antagonist tolvaptan (profiled below) may be tried. Two small studies have also suggested that the administration of albumin, which aids the expansion of plasma volume, may improve serum sodium concentration in patients with hypervolemic hyponatremia [34, 35]; however, these data must be replicated in larger studies.

Conventional therapy for treatment of ascites due to portal hypertension includes sodium restriction, diuretic therapy, and large-volume paracentesis [10]. The most effective diuretic combination consists of an aldosterone antagonist with potassium-sparing properties (e.g., spironolactone) plus a loop diuretic. Approximately 5–10 % of ascites cases are diuretic-resistant or diuretic-intractable, however, which portends a poor prognosis. The next step is pharmacologic treatment with a vasopressin receptor antagonist.

### Vasopressin Receptor Antagonists

Since AVP release is a primary cause of hyponatremia in cirrhosis, several vasopressin receptor antagonists have been evaluated in treating hyponatremia in patients with cirrhosis/ESLD and other conditions characterized by hypervolemic hyponatremia (e.g., heart failure) or euvolemic hyponatremia (e.g., the syndrome of inappropriate secretion of antidiuretic hormone [SIADH]). These include the intravenous dual V_1A_/V_2_-receptor antagonist conivaptan, and the oral V_2_-receptor antagonists lixivaptan (VPA-985), satavaptan, and tolvaptan. Currently, only conivaptan (described directly below) and tolvaptan (described in the following section) are approved for increasing serum sodium in patients with hypervolemic or euvolemic hyponatremia in the United States.

Conivaptan is administered via an intravenous loading dose of 20 mg followed by continuous infusion of 20 mg/day for 2–4 days (if serum sodium does not rise at the desired rate, it may be titrated upward to 40 mg over 24 h). In patients with Child-Pugh class A–C hepatic impairment, the recommended loading dose is 10 mg followed by a continuous infusion of 10 mg over 24 h for 2 to a maximum of 4 days (with titration up to 20 mg over 24 h if serum sodium is not rising at the desired rate) [36]. In a randomized, double-blind trial in 84 hospitalized patients with hypervolemic or euvolemic hyponatremia, conivaptan given as a 20-mg loading dose followed by a 96-h infusion of 40 or 80 mg/day significantly improved serum sodium levels compared with placebo, with efficacy evident after the first day of treatment. Area under the serum sodium concentration-time curve was significantly increased with both conivaptan doses versus placebo over 4 days (*P* < 0.0001 for both), and least-squares mean increases in serum sodium were 6.3 and 9.4 mEq/L versus 0.8 mEq/L with placebo. Conivaptan was generally well tolerated; four patients had to discontinue treatment due to injection site reactions [37]. A retrospective review of 24 cirrhotic patients with hyponatremia showed that conivaptan raised serum sodium in patients receiving or not receiving diuretics [38]. Because conivaptan is available only as a parenteral formulation, chronic use in cirrhotic patients with hyponatremia may be limited. Theoretically, it may not be advisable to use conivaptan in patients with cirrhosis because of the possibility that V_1A_ inhibition may result in splanchnic vasodilation [39]. The estimated cost of conivaptan (average wholesale price) is $573/day [40].

Lixivaptan (VPA-985) was shown to increase urine output, decrease urine osmolality, and increase serum osmolality and serum sodium in a dose-ascending single-dose study in patients with cirrhotic ascites [41], and to produce significant dose-related increases in water clearance and serum sodium in 44 hyponatremic patients with cirrhosis, heart failure, or SIADH [42]. In a randomized, double-blind trial in 60 hyponatremic patients with cirrhosis, lixivaptan 100 and 200 mg/day resulted in normalization of serum sodium (≥136 mEq/L) in 27 and 50 % of patients, respectively, compared with 0 % for placebo (*P* < 0.05 and *P* < 0.001, respectively), and also yielded significant reductions in urine osmolality and body weight [43].

Phase II trials of satavaptan suggested efficacy in controlling ascites in cirrhotic patients [44, 45]. However, in three randomized, double-blind phase III trials involving 1,200 patients with ascites, satavaptan was not shown to be more effective than placebo with respect to the primary endpoints (worsening of ascites in one study and cumulative number of paracenteses in the other two). In addition, while mortality rates for satavaptan and placebo were comparable in two of the studies, the third study reported a significant increase in mortality with satavaptan versus placebo (29.4 vs. 21.7 %, respectively; HR 1.47; 95 % CI 1.01–2.15; *P* = .049) [46]. Due to limited efficacy in treating the underlying causes and observable symptoms of hyponatremia, as well as accompanying safety concerns, the manufacturer discontinued development of this agent in 2008.

## Tolvaptan in Hypervolemic or Euvolemic Hyponatremia

Tolvaptan is an oral, selective vasopressin V_2_-receptor antagonist that blocks the effects of AVP, thus increasing free water excretion (aquaresis) and serum sodium concentration. Unlike diuretics, tolvaptan does not significantly affect urinary sodium or potassium excretion. It is currently indicated in the United States for treatment of clinically significant hypervolemic and euvolemic hyponatremia (serum sodium < 125 mEq/L) or less-marked hyponatremia that is symptomatic and resistant to correction by fluid restriction, including treatment of patients with cirrhosis, heart failure, or SIADH [11].

In an initial study, tolvaptan was shown to be more effective than fluid restriction in increasing serum sodium in 28 patients with euvolemic or hypervolemic hyponatremia (serum sodium < 135 mEq/L; tolvaptan, *n* = 17; fluid restriction, *n* = 11) [47]. Changes in serum sodium concentrations were +1.6 versus −0.8 mEq/L (*P* = 0.016) for tolvaptan versus fluid restriction, respectively, at 1 h after the first dose, +5.2 versus +0.7 mEq/L after 5 days (*P* = 0.019), and +5.7 versus +1.0 at the last visit (maximum, 27 days of treatment; *P* = 0.0065). In a study in 18 Japanese patients with intractable ascites or lower limb edema, in which hyponatremia (<120 mEq/L) was an exclusion criterion, tolvaptan doses of 15, 30, and 60 mg/day for 3 days dose-dependently decreased body weight and abdominal circumference and improved ascites and edema [48]. Mean changes in body weight after 3 days were −1.6, −2.6, and −3.4 kg for tolvaptan 15, 30, and 60 mg/day, respectively, and changes in abdominal circumference ranged from −2.8 to −6.0 cm. Urine osmolality was markedly decreased and remained decreased until the end of the study.

Tolvaptan was found to reverse euvolemic and hypervolemic hyponatremia (serum sodium < 135 mEq/L) in the Study of Ascending Levels of Tolvaptan in Hyponatremia (SALT)-1 and SALT-2 trials. In these two randomized, double-blind, placebo-controlled, phase 3 trials of similar design [12], a total of 448 patients were randomized to tolvaptan (*n* = 225; 15 mg once daily on day 1, increased at 1-day intervals to 30 and 60 mg/day if necessary during the first 4 days) or placebo (*n* = 223) in addition to standard therapy for 30 days. In total, 120 patients (27 %) had cirrhosis, 138 (31 %) had heart failure, and 190 (42 %) had SIADH or other causes of hyponatremia. Fluid restriction was avoided during the first 24 h of treatment to prevent overly rapid correction of serum sodium; 87 % of patients had no fluid restriction during this period [11]. Thereafter, fluid was restricted as clinically indicated (intake ≤ 1.5 L daily). Tolvaptan treatment resulted in a significantly greater increase in serum sodium at both time points (day 4 and 30) in both studies (*P* < 0.0001 for all comparisons). Pooled results for all patients showed changes in average daily serum sodium AUC of 4.0 for tolvaptan versus 0.4 mEq/L for placebo at day 4 and 6.2 versus 1.8 mEq/L at day 30 (both *P* < 0.0001) [11]. Overall, 14 % of tolvaptan versus 25 % of placebo patients (*P* < 0.01) required fluid restriction. A significantly greater increase in serum sodium was observed with tolvaptan treatment as early as 8 h after the first dose. Significant reductions in serum sodium at days 4 and 30 were also reported for patient subgroups with baseline sodium < 130 mEq/L and <125 mEq/L (both *P* < 0.0001). During a 7-day follow-up after stopping study treatment, serum sodium concentrations in the tolvaptan group decreased to levels similar to those in the placebo group.

**Fig. 5:**
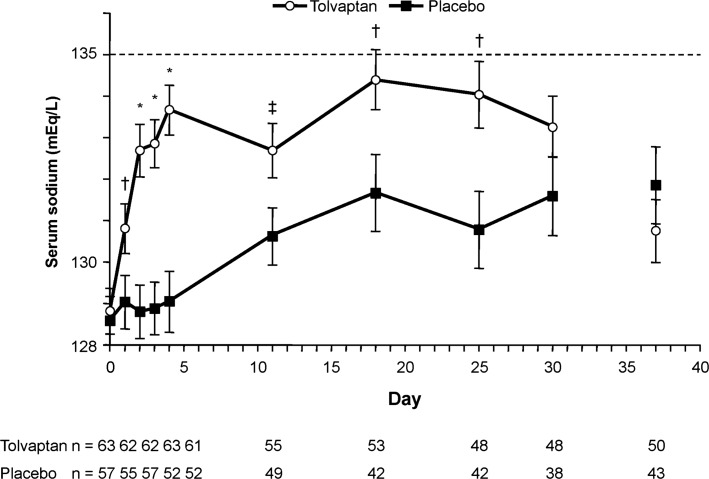
Observed serum sodium concentration throughout the study treatment period (days 1–30) and 7 days after stopping (day 37) in patients receiving tolvaptan or placebo. Error bars are ±SE (standard error of the mean). *P < 0.001, tolvaptan versus placebo; ^†^ *P* < 0.01, tolvaptan versus placebo; ^‡^ *P* < 0.05, tolvaptan versus placebo [49]

The SALT trials included 63 tolvaptan-treated patients and 57 placebo patients with cirrhosis [49]. The mean age for these patients (52 and 55 years, respectively) was younger than that for patients with heart failure or SIADH/other conditions (67 and 63, respectively) (data on file, Otsuka America Pharmaceutical, Inc., Rockville, MD). At baseline, 44 % had mild hyponatremia (serum sodium 130–134 mEq/L), 56 % had marked hyponatremia (serum sodium < 130 mEq/L), 85 % had cirrhosis due to alcohol and/or hepatitis B/C, and 80 % were Child-Pugh class B/C. Changes in average daily serum sodium AUC for the SALT patients with cirrhosis were 3.5 mEq/L for tolvaptan versus 0.3 mEq/L for placebo at day 4 and 4.2 mEq/L versus 1.2 mEq/L at day 30 (both *P* < 0.0001). Patients receiving tolvaptan had significantly greater increases in serum sodium (*P* < 0.05) as early as 8 h after the first dose and at days 2, 3, 4, 11, 18, and 25 (Fig. 5). The proportion of patients with normalized serum sodium was significantly greater with tolvaptan at day 4 (41 vs. 11 %, *P* = 0.0002) and numerically greater at day 30 (33 vs. 19 %, *P* = 0.08). Mean mental component summary scores of the Medical Outcomes Study 12-item Short Form General Health Survey (SF-12) improved from baseline to day 30 in the tolvaptan group but not the placebo group (4.68 vs. 0.08, *P* = 0.02). Before the study began, 98 % of the tolvaptan group were taking diuretics (with the majority on a moderate dose: spironolactone < 200 mg/day or furosemide < 80 mg/day); only 6 % discontinued diuretic use during the trials. Diuretic use did not appear to have an impact on treatment with tolvaptan [49]; however, the data from this limited analysis need to be confirmed in larger clinical trials.

In the open-label SALTWATER study, 111 patients (94 with serum sodium < 135 mEq/L) who had previously received tolvaptan or placebo in the SALT trials were treated with tolvaptan after having returned to standard care for at least 7 days. Average serum sodium levels in these patients increased to approximately the same levels as those observed with tolvaptan treatment in the SALT trials and were maintained for at least 1 year [11, 50].

Adverse events in the SALT trials were generally similar in the tolvaptan and placebo groups [12]. The most common adverse events in tolvaptan patients were thirst (14 % with tolvaptan vs. 5 % with placebo) and dry mouth (13 vs. 4 %, respectively). Desirable rates of sodium correction (>0.5 mEq/L/h) were exceeded in 1.8 % of 223 tolvaptan patients during the 24 h after the first dose. The range of desirable increase was exceeded (>146 mEq/L) in 4 (1.8 %) of the tolvaptan patients.

In the analysis of cirrhotic patients in the SALT trials, gastrointestinal bleeding was reported in six out of 63 (10 %) tolvaptan-treated patients and one out of 57 (2 %) placebo-treated patients (*P* = 0.11) [49]. Among the tolvaptan recipients, five had evidence of upper gastrointestinal hemorrhage and concomitant esophageal varices and one had a self-limited episode of bright red blood per rectum that was attributed to hemorrhoids. The placebo patient had a gingival hemorrhage and concomitant esophageal varices that were not considered to be the cause of bleeding. The investigators suggested that these events were likely related to portal hypertension and esophageal varices, but that this could not be established based on the event descriptions, and noted that thus far there are no known mechanisms by which tolvaptan would increase the risk for variceal bleeding. The overall safety of tolvaptan in patients with cirrhosis remains to be confirmed in larger, prospective, placebo-controlled trials. Tolvaptan should be used in cirrhotic patients only when the need to treat outweighs the risk of gastrointestinal bleeding.

## Use of Tolvaptan in Patients with Cirrhosis/ESLD

Tolvaptan is indicated for the treatment of clinically significant hypervolemic or euvolemic hyponatremia characterized by serum sodium < 125 mEq/L or by less-marked symptomatic hyponatremia that has resisted correction with fluid restriction [11]. Tolvaptan is contraindicated in patients with hypovolemic hyponatremia, in those unable to sense or respond appropriately to thirst, in those who require an urgent increase in serum sodium to prevent or treat serious neurologic symptoms, in anuric patients, and in patients taking strong inhibitors of CYP3A4 isoenzymes. Tolvaptan should be used only when the need to treat outweighs the risk, especially given that data are currently available for short-term treatment only.

It is recommended that tolvaptan therapy be initiated (and re-initiated) in the hospital setting, with frequent monitoring of the patient’s serum sodium level to ensure that overly rapid correction does not occur. Based on the pharmacokinetics of tolvaptan, serum sodium should be monitored within the first 8–12 h following initiation, and then every 24 h thereafter. We recommend the dosing paradigm used in the SALT trials, beginning with 15 mg once daily, and increasing the dose at 24-h intervals to 30 mg once daily and then to 60 mg once daily if serum sodium is not raised to the desired level [12]. No adjustments are necessary based on age, gender, race, cardiac function, hepatic function, or renal function (providing creatinine clearance is ≥10 mL/min). Tolvaptan may be taken without regard to the timing of meals. Fluid restriction should be avoided during the first 24 h of treatment to prevent overly rapid correction of serum sodium, and patients should be advised to drink if they are thirsty. Once tolvaptan therapy is completed, fluid restriction or other therapies can be resumed, and changes in serum sodium and volume status should be monitored.

If hyponatremia recurs after tolvaptan therapy has been stopped, tolvaptan can be re-started in a hospital setting; readmission may not be necessary (i.e., a step-down unit may be utilized), provided adequate provisions are made to monitor treatment responses and prevent overly rapid correction of serum sodium levels. Among patients previously enrolled in the SALT trials, re-treatment with tolvaptan raised serum sodium to levels similar to those achieved during initial therapy, and levels were maintained during long-term daily treatment [50]. The estimated cost of tolvaptan (average wholesale price) is $300/day [40].

## Conclusions

Hyponatremia is common in patients with cirrhosis and ESLD and is associated with increased risk of morbidity and mortality. Mortality is significant in patients on liver transplant waiting lists and correlates with hyponatremia. Adjusting MELD scoring for hyponatremia improves the prediction of mortality in waiting list patients and may result in more appropriate allocation of grafts.

The selective oral vasopressin V_2_-receptor antagonist tolvaptan acts to increase free water excretion and is effective in resolving hyponatremia in patients with cirrhosis and in others with hypervolemic or euvolemic hyponatremia. Tolvaptan and other vasopressin antagonists have the potential to improve outcomes in these patient populations.

## Abbreviations

ADH: Antidiuretic hormone
APACHE: Acute Physiology and Chronic Health Evaluation
AUC: Area under the curve
AVP: Arginine vasopressin
CAPPS: Cirrhotic Ascites Patient Population Survey
CTP: Child-Turcotte-Pugh
ESLD: End-stage liver disease
ICU: Intensive care unit
MELD: Model for end-stage liver disease
MESO: MELD-sodium
MOLTO: Models for Optimal Liver Transplant Outcomes
Na: Sodium
NIDDK-LTD: National Institute of Diabetes and Digestive and Kidney Diseases Liver Transplantation Database
OR: Odds ratio
SALT: Study of Ascending Levels of Tolvaptan in Hyponatremia
SALTWATER: The Safety and sodium Assessment of Long-term Tolvaptan With hyponatremia: A year-long, open-label Trial to gain Experience under Real-world conditions
SF-12: Medical Outcomes Study 12-item Short Form General Health Survey
SIADH: Syndrome of inappropriate secretion of antidiuretic hormone
SOFA: Sequential Organ Failure Assessment
